# Pathogenicity of H5N8 High Pathogenicity Avian Influenza Virus in Chickens and Ducks from South Korea in 2020–2021

**DOI:** 10.3390/v13101903

**Published:** 2021-09-23

**Authors:** Min-Ji Park, Ra Mi Cha, Soo-Jeong Kye, Yu-Na Lee, Na-Yeong Kim, Yoon-Gi Baek, Gyeong-Beom Heo, Mingeun Sagong, Kwang-Nyeong Lee, Youn-Jeong Lee, Eun-Kyoung Lee

**Affiliations:** Avian Influenza Research & Diagnostic Division, Animal and Plant Quarantine Agency, 177 Hyeoksin 8-ro, Gimcheon-si 39660, Gyeongsangbuk-do, Korea; vksldpf4535@gmail.com (M.-J.P.); rami.cha01@korea.kr (R.M.C.); cessna70@korea.kr (S.-J.K.); ynlee27@korea.kr (Y.-N.L.); kny-6099@naver.com (N.-Y.K.); zigindagoma@gmail.com (Y.-G.B.); imheo@korea.kr (G.-B.H.); sagongmg@korea.kr (M.S.); leekwn@korea.kr (K.-N.L.); leeyj700@korea.kr (Y.-J.L.)

**Keywords:** high pathogenicity avian influenza, pathogenicity, chicken, ducks

## Abstract

During the 2020–2021 winter season, an outbreak of clade 2.3.4.4b H5N8 high pathogenicity avian influenza (HPAI) virus occurred in South Korea. Here, we evaluated the pathogenicity and transmissibility of A/mandarin duck/Korea/H242/2020 (H5N8) (H242/20(H5N8)) first isolated from this outbreak in specific pathogen-free (SPF) chickens and commercial ducks in comparison with those of A/duck/Korea/HD1/2017(H5N6) (HD1/17(H5N6)) from a previous HPAI outbreak in 2017–2018. In chickens, the 50% chicken lethal dose and mean death time of H242/20(H5N8) group were 10^4.5^ EID_50_ and 4.3 days, respectively, which indicate less virulent than those of HD1/17(H5N6) (10^3.6^ EID_50_ and 2.2 days). Whereas, chickens inoculated with H242/20(H5N8) survived longer and had a higher titer of viral shedding than those inoculated with HD1/17(H5N6), which may increase the risk of viral contamination on farms. All ducks infected with either HPAI virus survived without clinical symptoms. In addition, they exhibited a longer virus shedding period and a higher transmission rate, indicating that ducks may play an important role as a silent carrier of both HPAI viruses. These results suggest that the pathogenic characteristics of HPAI viruses in chickens and ducks need to be considered to effectively control HPAI outbreaks in the field.

## 1. Introduction

Since 1997, high pathogenicity avian influenza (HPAI) H5 viruses of the A/goose/Guangdong/1/1996 (Gs/GD) lineage have caused outbreaks in countries worldwide and have evolved continuously into diverse clades and subgroups. Starting 2014 onwards, H5Nx HPAI viruses belonging to clade 2.3.4.4., including H5N2, H5N6, and H5N8, have circulated globally in wild birds and poultry, causing huge economic losses in the poultry industry [1,2].

In South Korea, HPAI outbreaks have occurred due to the introduction of HPAI viruses belonging to multiple clades and with diverse genotypes. During 2014–2016, clade 2.3.4.4c H5N8 HPAI virus caused a fifth outbreak, which followed four previous outbreaks of H5N1 viruses in 2003–2011 [3,4]. In the 2016–2017 winter season, the largest HPAI outbreaks were due to newly introduced clade 2.3.4.4e H5N6 viruses. Subsequently, clade 2.3.4.4b H5N8 viruses also caused an outbreak [5,6]. In 2017–2018, a H5N6 virus from different subclade (clade 2.3.4.4b) caused an outbreak on poultry farms [7,8]. The pathogenicity and transmissibility of HPAI viruses belonging to clade 2.3.4.4 vary according to genetic differences [3,6,9,10,11,12,13,14].

In October 2020, clade 2.3.4.4b H5N8 virus was first isolated from wild birds and was identified in a domestic duck farm in Korea one month later. From the winter of 2020–2021 until April 2021, 109 outbreaks in poultry and 234 cases in wild birds reported (based on data from Korean Animal Health Integrated System (KAHIS)). H5N8 HPAI viruses from the 2020–2021 outbreak revealed to have at least seven genotypes [15]. The H5N8 HPAI virus that first circulated during the 2020–2021 outbreak in Korea is genetically close to viruses reported in Europe during 2019–2020 [15,16,17,18]. Major H5N8 viruses that circulated later during the 2020–2021 outbreak have a close genetic relationship with viruses detected in Europe in late 2020 [15].

In this study, we investigated the pathogenicity and transmissibility of A/mandarin duck/Korea/H242/2020(H5N8)(H242/20(H5N8)), which was first isolated during the 2020–2021 HPAI outbreak, in specific pathogen-free (SPF) chickens and commercial ducks.

## 2. Materials and Methods

### 2.1. Viruses

A/mandarin duck/Korea/H242/2020(H5N8) (H242/20(H5N8)) was isolated from fecal samples of mandarin ducks in October 2020 and was the first reported detection of H5N8 during the 2020–2021 HPAI outbreak in Korea [15]. Its pathogenicity was compared with that of A/duck/Korea/HD1/2017(H5N6) (HD1/17(H5N6)), which was isolated from a commercial duck farm during the 2017–2018 HPAI outbreak in Korea [7]. Working stocks were titrated in specific pathogen-free (SPF) chickens eggs using standard methods at the time of isolation and stored at −70 °C until further use.

### 2.2. Animals and Housing

Forty-six five-week-old SPF chickens (Gallus gallus domesticus) were obtained from Namduck Sanitec (Icheon, Gyeonggi-do, Korea). Thirty-six two-week-old ducks were obtained from a commercial duck farm. Chicken and duck serum samples were tested before experiments using a competitive enzyme-linked immunosorbent assay (AniGen AIV Ab ELISA kit; Bionote, Suwon, South Korea) to confirm that all birds were free from antibodies against avian influenza (AI). The birds were housed in negative-pressure, high efficiency, air-filtered isolation cabinets within a biosafety level 3 facility. Water and feed were provided ad libitum. 

### 2.3. Experimental Design

To assess pathogenicity, an intravenous pathogenicity index (IVPI) test with SPF chickens was performed according to the instructions in the World Organization of Animal Health (OIE) manual [19]. Briefly, ten 5-week-old SPF chickens were intravenously inoculated with 0.1 mL of a 1:10 dilution of bacteria-free allantoic fluid containing H242/20(H5N8) or HD1/17(H5N6). To evaluate the mean chicken lethal dose (cLD_50_), chickens were divided into four groups (*n* = 5) and intranasally inoculated with 0.1 mL of serial 10-fold dilutions, ranging from 10^3^ to 10^6^ mean egg infectious dose (EID_50_), of H242/20(H5N8) or HD1/17(H5N6). To examine mean bird infectious dose (BID_50_), ducks were divided into three group (*n* = 5) and intranasally inoculated with 0.1 mL of 10^2^, 10^4^, and 10^6^ EID_50_ of H242/20(H5N8) or HD1/17(H5N6). Serum were collected at 17 days post inoculation (dpi) and hemagglutination inhibition (HI) test were performed. EID_50_ and BID_50_ were calculated using the method of Reed and Muench [20]. To determine virus transmissibility in chickens and ducks, each bird was intranasally inoculated with 0.1 mL of 10^6^ EID_50_ of H242/20(H5N8) or HD1/17(H5N6). Eight hours later, three naïve chickens or ducks were co-housed with the inoculated group. Three more chickens or ducks were inoculated intranasally with 10^6^ EID_50_/0.1 mL of each virus to examine viral replications in internal organs at 3 dpi. All animal experiments were reviewed and approved by the Institutional Animal Care and Use Committee of the Animal and Plant Quarantine Agency (APQA) (approval no: 2019-490).

### 2.4. Viral Shedding and Replication in Internal Organs

Oropharyngeal (OP) and cloacal (CL) swabs were collected at 1, 2, 3, 4, 5, 6, 7, 10, and 14 dpi in the 10^6^ EID_50_-inoculated and contact groups to evaluate viral shedding. After birds died or were euthanized at 3 dpi, three birds from each inoculated group were necropsied, 12 organs (trachea, thymus, heart, lung, kidney, brain, pancreas, cecal tonsil, liver, spleen, muscle, and proventriculus) were collected, and virus replication in each organ was assessed. For virus isolation, each OP and CL swab sample was suspended in 1 mL of Dulbecco’s Modified Eagle Medium (Gibco; Invitrogen, Carlsbad, CA, USA) containing antibiotics (Antibiotic Antimycotic; Invitrogen), and each tissue sample was homogenized (wt/vol ratio of 10%). Samples were then centrifuged at 3500 rpm for 5 min. The supernatant was titrated in chicken embryo fibroblasts (DF-1) to determine the 50% tissue culture infective dose (TCID_50_). The virus titer was calculated using the method of Reed and Muench [20]. The limit of virus detection was <1 log_10_ TCID_50_/0.1 mL. Student’s *t*-test for independent sample was performed using Graphpad Prism 5 (San Diego, CA, USA).

### 2.5. Hemagglutination Inhibition (HI) Test 

The HI test was performed using standard procedures according to the OIE manual with serum samples from remaining live birds at 17 dpi. Briefly, serum samples were treated with receptor-destroying enzyme (RDEII, Denka Seiken, Japan) to remove non-specific inhibitors following manufacture’s instruction (serum: enzyme = 1:4). Treated serum was serially diluted 2-fold in v-bottom 96 well plate and 8 HA unit of homologous virus strain antigen was used for the HI test. Positive and negative control serum were tested in each plate. Back titration of 8 HA unit of virus antigen was also performed.

## 3. Results and Discussion

We evaluated the pathogenicity and transmissibility of H242/20(H5N8) in SPF chickens and ducks. We compared these findings with those of HD1/17(H5N6), a virus belonging to the same clade 2.3.4.4b but a different subtype that was isolated during a previous HPAI outbreak in 2017–2018 in Korea. 

The IVPI values of H242/20(H5N8) and HD1/17(H5N6) were 2.88 and 2.98, respectively, confirming that both viruses are classified as HPAI according to the OIE standard (Table 1) [19]. Both viruses caused death in a dose-dependent manner; the cLD_50_ of H242/20(H5N8) and HD1/17(H5N6) was 10^4.5^ and 10^3.6^ EID_50_, respectively. These results indicate that an approximately 10 times higher dose of H242/20(H5N8) than of HD1/17(H5N6) is required to kill SPF chickens. A viral dose of 10^6^ EID_50_ of H242/20(H5N8) resulted in 100% mortality in chickens, with a mean death time (MDT) of 4.3 days. In the contact group, one of three SPF chickens died, demonstrating that transmissibility was 33.3%. Meanwhile, the MDT of HD1/17(H5N6)-inoculated chickens was 2.2 days, which was shorter than that of H242/20(H5N8)-inoculated chickens (Table 1). None of the surviving SPF chickens showed the HI response.

**Figure 1:**
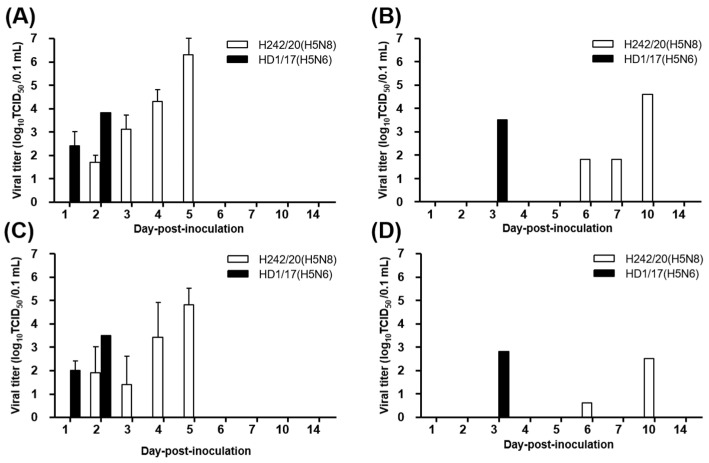
Virus isolation from oropharyngeal (OP) (**A**,**B**) and cloacal (CL) (**C**,**D**) swab samples of virus-exposed chickens. Five chickens were intranasally inoculated with 10^6^ EID_50_/0.1 mL H242/20(H5N8) or HD1/17(H5N6) (**A**,**C**). For the contact group, three chickens were co-housed with H242/20(H5N8) or HD1/17(H5N6)-infected chickens (**B**,**D**). Viral titers are shown as the mean ± standard deviation.

**Figure 2:**
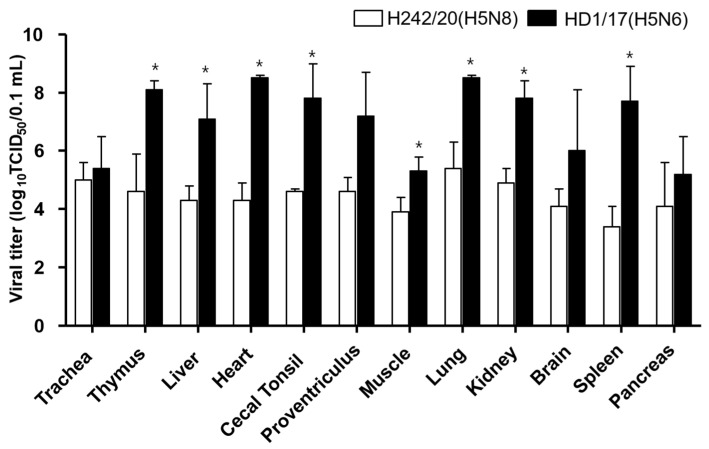
Virus titer of H242/20(H5N8) virus and HD1/17(H5N6) virus in various organs from SPF chickens which were inoculated intranasally with a viral dose of 10^6^ EID_50_. H242/20(H5N8) and HD1/17(H5N6) virus infected organ tissues were collected at 3 dpi. The virus titers of the organ tissues (10% homogenates) were measured in chicken embryo fibroblast cell (DF-1). Data shown indicate the average of calculable positive organ titer. *; *p* < 0.05.

In H242/20(H5N8)-inoculated SPF chickens, viral shedding was observed via the OP and CL routes from 2 to 5 dpi and the titer peaked at 5 dpi in OP and CL samples at 10^4.8–6.3^ TCID_50_/0.1 mL (Figure 1 and Figure S1). In contact birds, a single chicken in each virus showed viral shedding via OP and CL route and found dead (Figure 1 and Table 1). In HD1/17(H5N6)-inoculated chickens, viral shedding started at 1 dpi and lasted until 2 dpi before death occurred, with peak titers of 10^3.5–3.8^ TCID_50_/0.1 mL in both OP and CL samples (Figure 1 and Figure S1). H242/20(H5N8)-inoculated chickens survived longer and exhibited a higher titer of viral shedding than HD1/17(H5N6)-inoculated chickens. When virus replication was measured in organs at 3 dpi, the virus was isolated from all tested tissues, indicating that both viruses caused systemic infection in chickens (Figure 2 and Table S1). Notably, viral titers in organs were much lower in H242/20(H5N8)-inoculated chickens (10^3.4–5.4^ TCID_50_/0.1 mL) than in HD1/17(H5N6)-inoculated chickens (10^5.4–8.3^ TCID_50_/0.1 mL) at 3 dpi. These findings also suggest that the peak of viral titer in organs may have reached 3 dpi later in H242/209(H5N8) inoculated chickens. 

In summary, H242/20(H5N8) was less virulent than HD1/17(H5N6), as demonstrated by its higher cLD_50_, longer MDT/viral shedding period, and lower viral titers in organs at 3 dpi. Meanwhile, these results suggest that the risk of viral contamination on farms may be increased by H242/20(H5N8), which has a long viral shedding period and MDT in chickens. 

Since 2014, several outbreaks of clade 2.3.4.4 H5N8 and H5N6 HPAI viruses have occurred in Korea. Pathogenicity differs between the representative strain of each HPAI outbreak [6,10,11,12,21]. H242/20(H5N8) in this study has similar pathogenic features as a H5N8 virus isolated in 2014 (Buan2/14(H5N8)), which has a high cLD_50_ (10^5.3^ EID_50_) and long MDT (4.5 days) in SPF chickens [12]. The reduced virulence of the Buan2/14(H5N8) virus may lead to late recognition on farms and sustained outbreaks, which may be one reason why outbreaks of this virus lasted almost 2 years (2014–2016) [10]. The experimental pathogenic features of H242/20(H5N8), which is similar to Buan2/14(H5N8), indicate that there is a risk of sustained outbreaks without active surveillance and adequate control measures in the field. Therefore, the pathogenic features of HPAI viruses in experimental settings needs to be considered for effective HPAI control measures in the field.

From December 2019 to June 2020, clade 2.3.4.4b HPAI H5N8 viruses were detected in wild birds and poultry in European countries [16,17,18,22,23]. H242/20(H5N8) in this study is genetically similar to these European viruses detected in 2019–2020 [15]. In addition, H242/20(H5N8) shows genetic similarity with H5N8 HPAI viruses isolated in Japan during a recent outbreak in 2020–2021 [24,25,26]. The genetic similarity between HPAI viruses may correlate with similar pathogenicity in chickens. The pathogenicity of H5N8/2020 isolated in Japan and reported in a recent study, which has a cLD_50_ of 10^4.63^ EID_50_ and MDT of 5.6 days, is similar to our findings concerning H242/20(H5N8) in SPF chickens [24].

**Figure 3:**
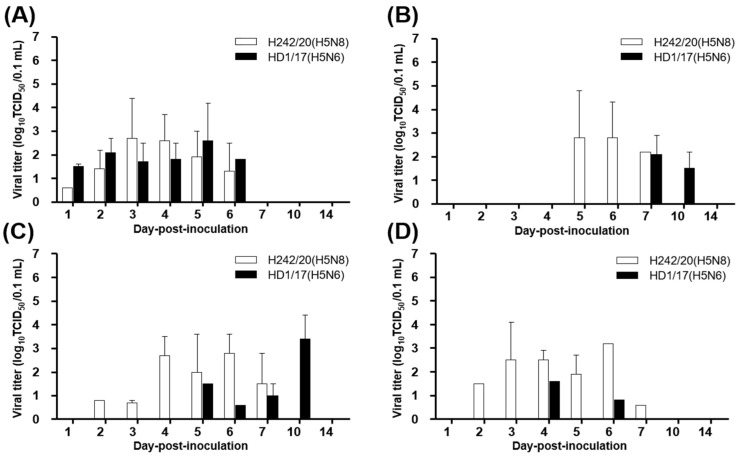
Virus isolation from oropharyngeal (OP) (**A**,**B**) and cloacal (CL)(**C**,**D**) swab samples of virus-exposed ducks. Ducks (*n* = 5) were intranasally inoculated with 10^6^ EID_50_/0.1 mL H242/20(H5N8) or HD1/17(H5N6) (**A**,**C**). For the contact group, naïve ducks (*n* = 3) were co-housed with H242/20(H5N8)- or HD1/17(H5N6)-infected ducks (**B**,**D**). Viral titers are shown as the mean ± standard deviation.

**Figure 4:**
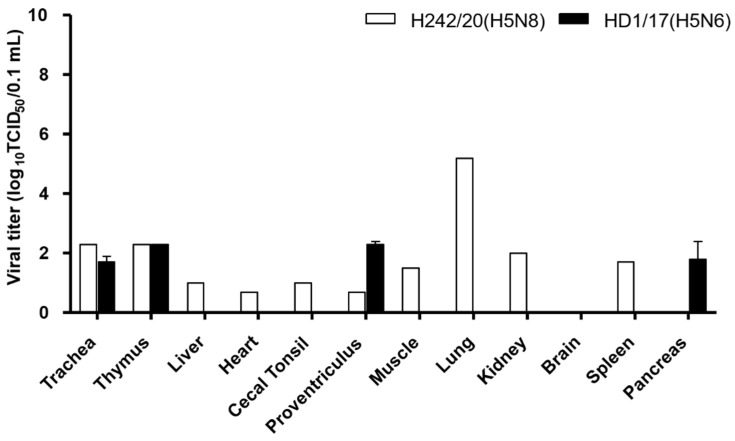
Virus titer of H242/20(H5N8) virus and HD1/17(H5N6) virus in various organs from ducks, which were inoculated intranasally with a viral dose of 10^6^ EID_50_. H242/20(H5N8) virus and HD1/17(H5N6) virus infected organ tissues were collected at 3 dpi. The virus titers of the organ tissues (10% homogenates) were measured in chicken embryo fibroblast cell (DF-1). Data shown indicate the average of calculable positive organ titer.

The pathogenicity of H242/20(H5N8) was evaluated in commercial ducks and compared with that of HD1/17(H5N6). All ducks inoculated with either virus survived without clinical symptoms. The mean bird infectious dose (BID_50_) of H242/20(H5N8) and HD1/17(H5N6) in ducks was 10^5.0–5.3^ EID_50_, indicating that a higher dose of virus is required to cause infection in ducks than in chickens. In addition, 10^4^ dose group, both virus infected chickens showed some mortality but no seroconversion observed in ducks. Ducks infected with either virus exhibited viral shedding via the OP and CL routes until 6 and 10 dpi, respectively. In contact groups, all ducks shed the virus until 10 dpi. Whereas, the peak titer of viral shedding was lower than in chickens (Figure 3). Only one of three ducks tested positive for virus replication following intranasal infection of 10^6^ EID_50_ of H242/20(H5N8), and the viral titers in organs were much lower in ducks than in SPF chickens (Figure 4 and Table S2). Virus-infected ducks in 10^6^ dose group showed seroconversion (Table 2).

Ducks inoculated with H242/20(H5N8) survived without clinical signs and exhibited long viral shedding and a 100% transmission rate. These pathogenic features of the HPAI virus in ducks may contribute to their role as a silent carrier in the field. Bayesian phylodynamic analysis of H5N8 viruses from the 2020–2021 outbreak also revealed that HPAI viruses initially disseminate from migratory waterfowl to domestic ducks, and then domestic ducks most likely contribute to transmission of the viruses to chickens and other minor poultry [15].

Our findings in ducks are consistent with those concerning H5N8 HPAI viruses from the 2014–2016 outbreak. An abundant amount of viral shedding without clinical signs in ducks may be one of the reasons for longer outbreaks [3]. Additionally, genetic analysis demonstrated that H5N8 viruses in the 2014–2016 outbreak was likely to transmit from wild birds to domestic ducks, and this played a central role in virus spread to domestic poultry [13,27]. A recent study about comparative pathogenicity of HPAI H5 viruses in Netherland also suggested that virus shedding of duck species including both domestic and wild ducks might increase transmission to the poultry sector [28]. Collectively, our results in ducks, as well as previous findings concerning Korean HPAI outbreaks, indicate that domestic ducks play an important role in transmission of H5N8 HPAI viruses in the field.

In this study, H242/20(H5N8) from the 2020–2021 outbreak was less virulent than HD1/17(H5N6) in chickens. Virus-infected ducks survived without clinical signs and shed the virus for longer and showed a higher transmission rate than chickens, indicating that ducks play an important role as a silent carrier on farms. The pathogenicity of HPAI viruses differs between hosts and may affect virus spread in the field. Therefore, the pathogenic features of HPAI viruses experimentally evaluated in chickens and ducks, need to be considered for the effective control measure of HPAI outbreaks in the field.

## Figures and Tables

**Table 1:** Pathogenicity and transmissibility of H242/20(H5N8) and HD1/17(H5N6) in SPF chickens.

Virus	IVPI	cLD_50_ (EID_50_)	Virus Dose (EID_50_/0.1 mL)	Mortality (%)	MDT (Day)	HI Titer ^a^ (log_2_, Mean ± SD)
A/mandarin duck/ Korea/H242/2020 (H5N8)	2.88	10^4.5^	10^6^	5/5 (100)	4.3	NT
			10^5^	4/5 (80)	5.2	-(0/1)
			10^4^	1/4 ^(1)^ (25)	7.4	-(0/3)
			10^3^	0/5 (0)	-	-(0/5)
			Contact	1/3 (33)	10	-(0/2)
A/duck/Korea/ HD1/2017 (H5N6)	2.98	10^3.6^	10^6^	5/5 (100)	2.2	NT
			10^5^	5/5 (100)	4.8	NT
			10^4^	4/5 (80)	5.7	-(0/1)
			10^3^	0/5 (0)	-	-(0/5)
			Contact	1/2 ^(2)^ (50)	7	-(0/1)

The number of affected birds/birds per group were shown. SPF chickens were intranasally inoculated serial 10-fold dilutions, ranging from 10^3.0^ to 10^6.0^ EID_50_/0.1 mL of H242/20(H5N8) and HD1/17(H5N6). IVPI, intravenous pathogenicity index; cLD_50_, mean chicken lethal dose; MDT, mean death time; NT, not tested. ^(1)^ Early death occurred in one SPF chicken, possibly caused by unidentified complication, followed by HPAI infection. Therefore, this bird was excluded in the measurement of cLD_50_. ^(2)^ One of the three birds in the contact group was sacrificed on 10 dpi due to the leg injury. ^a^ The sera were sampled at 17 dpi.

**Table 2:** Pathogenicity and transmissibility of H242/20(H5N8) and HD1/17(H5N6) in ducks.

Virus	Virus Dose (EID_50_/0.1 mL)	Mortality (%)	HI Titer ^a^ (log_2_, Mean ± SD)	BID_50_ (EID_50_)
A/mandarin duck/ Korea/H242/2020 (H5N8)	10^6^	0/5 (0)	3.0 ± 1.4 (4/5)	10^5.3^
	10^4^	0/5 (0)	-(0/5)	
	10^2^	0/5 (0)	-(0/5)	
	Contact	0/3 (0)	2.7 ± 0.6 (3/3)	
A/duck/Korea/ HD1/2017 (H5N6)	10^6^	0/5 (0)	2.3 ± 1.3 (5/5)	10^5.0^
	10^4^	0/5 (0)	-(0/5)	
	10^2^	0/5 (0)	-(0/5)	
	Contact	0/3 (0)	3.0 ± 1.7 (3/3)	

The number of affected birds/birds per group were shown. Ducks were intranasally inoculated ranging from 10^2^, 10^4^, 10^6^ EID_50_/0.1 mL of H242/20(H5N8) and HD1/17(H5N6), BID_50_, mean bird infectious dose. ^a^ The sera were sampled at 17 dpi.

## Supplementary Materials

The following are available online at https://www.mdpi.com/article/10.3390/v13101903/s1, Figure S1: Survival curve of chickens inoculated with H242/20(H5N8) and HD1/17(H5N6). Table S1: Virus titers in organs of individual chickens inoculated with H242/20(H5N8) and HD1/17(H5N6). Table S2: Virus titers in organs of individual ducks inoculated with H242/20(H5N8) and HD1/17(H5N6).

## References

[1] Poovorawan Y., Pyungporn S., Prachayangprecha S., Makkoch J. (2013). Global alert to avian influenza virus infection: From H5N1 to H7N9. Pathog. Glob. Health.

[2] Lee D.-H., Criado M.F., Swayne D.E. (2021). Pathobiological Origins and Evolutionary History of Highly Pathogenic Avian Influenza Viruses. Cold Spring Harb. Perspect. Med..

[3] Song B.-M., Lee E.-K., Lee Y.-N., Heo G.-B., Lee H.-S., Lee Y.-J. (2017). Phylogeographical characterization of H5N8 viruses isolated from poultry and wild birds during 2014–2016 in South Korea. J. Vet. Sci..

[4] Lee D.-H., Bertran K., Kwon J.-H., Swayne D.E. (2017). Evolution, global spread, and pathogenicity of highly pathogenic avian influenza H5Nx clade 2.3.4.4. J. Vet. Sci..

[5] Lee E.-K., Song B.-M., Lee Y.-N., Heo G.-B., Bae Y.-C., Joh S.-J., Park S.-C., Choi K.-S., Lee H.-J., Jang I. (2017). Multiple novel H5N6 highly pathogenic avian influenza viruses, South Korea, 2016. Infect. Genet. Evol..

[6] Park S.C., Song B.M., Lee Y.N., Lee E.K., Heo G.B., Kye S.J., Lee K.H., Bae Y.C., Lee Y.J., Kim B. (2019). Pathogenicity of clade 2.3.4.4 H5N6 highly pathogenic avian influenza virus in three chicken breeds from South Korea in 2016/2017. J. Vet. Sci..

[7] Lee E.-K., Lee Y.-N., Kye S.-J., Lewis N.S., Brown I.H., Sagong M., Heo G.-B., Kang Y.-M., Cho H.-K., Kang H.-M. (2018). Characterization of a novel reassortant H5N6 highly pathogenic avian influenza virus clade 2.3.4.4 in Korea, 2017. Emerg. Microbes Infect..

[8] Baek Y.-G., Lee Y.-N., Lee D.-H., Cheon S.-H., Kye S.-J., Park Y.-R., Si Y.-J., Lee M.-H., Lee Y.-J. (2020). A novel reassortant clade 2.3.4.4 highly pathogenic avian influenza H5N6 virus identified in South Korea in 2018. Infect. Genet. Evol..

[9] Jeong J., Kang H.-M., Lee E.-K., Song B.-M., Kwon Y.-K., Kim H.-R., Choi K.-S., Kim J.-Y., Lee H.-J., Moon O.-K. (2014). Highly pathogenic avian influenza virus (H5N8) in domestic poultry and its relationship with migratory birds in South Korea during 2014. Vet. Microbiol..

[10] Lee E.-K., Song B.-M., Kang H.-M., Woo S.-H., Heo G.-B., Jung S.C., Park Y.H., Lee Y.-J., Kim J.-H. (2016). Experimental infection of SPF and Korean native chickens with highly pathogenic avian influenza virus (H5N8). Poult. Sci..

[11] Kang H.-M., Lee E.-K., Song B.-M., Jeong J., Choi J.-G., Jeong J., Moon O.-K., Yoon H., Cho Y., Kang Y.-M. (2015). Novel Reassortant Influenza A(H5N8) Viruses among Inoculated Domestic and Wild Ducks, South Korea, 2014. Emerg. Infect. Dis..

[12] Song B.-M., Kang H.-M., Lee E.-K., Youn-Jeong L., Kang Y., Lee H.-S., Lee Y.-J. (2015). Pathogenicity of H5N8 virus in chickens from Korea in 2014. J. Vet. Sci..

[13] Kwon J., Bahl J., Swayne D.E., Lee Y., Lee Y., Song C., Lee D. (2019). Domestic ducks play a major role in the maintenance and spread of H5N8 highly pathogenic avian influenza viruses in South Korea. Transbound. Emerg. Dis..

[14] Jeong O.-M., Kim M.-J., Kang H.-M., Kim H.-R., Kim Y.-J., Joh S.-J., Kwon J.-H., Lee Y.-J. (2009). Experimental infection of chickens, ducks and quails with the highly pathogenic H5N1 avian influenza virus. J. Vet. Sci..

[15] Baek Y.-G., Lee Y.-N., Lee D.-H., Shin J.-I., Lee J.-H., Chung D., Lee E.-K., Heo G.-B., Sagong M., Kye S.-J. (2021). Multiple Reassortants of H5N8 Clade 2.3.4.4b Highly Pathogenic Avian Influenza Viruses Detected in South Korea during the Winter of 2020–2021. Viruses.

[16] King J., Schulze C., Engelhardt A., Hlinak A., Lennermann S.-L., Rigbers K., Skuballa J., Staubach C., Mettenleiter T.C., Harder T. (2020). Novel HPAIV H5N8 Reassortant (Clade 2.3.4.4b) Detected in Germany. Viruses.

[17] Świętoń E., Fusaro A., Shittu I., Niemczuk K., Zecchin B., Joannis T., Bonfante F., Śmietanka K., Terregino C. (2020). Sub-Saharan Africa and Eurasia Ancestry of Reassortant Highly Pathogenic Avian Influenza A(H5N8) Virus, Europe, December 2019. Emerg. Infect. Dis..

[18] Lewis N.S., Banyard A.C., Whittard E., Karibayev T., Al Kafagi T., Chvala I., Byrne A., Akberovna S.M., King J., Harder T. (2021). Emergence and spread of novel H5N8, H5N5 and H5N1 clade 2.3.4.4 highly pathogenic avian influenza in 2020. Emerg. Microbes Infect..

[19] OIE Manual of Diagnostic Tests and Vaccines for Terrestrial Animals. https://www.oie.int/fileadmin/Home/eng/Health_standards/tahm/3.03.04_AI.pdf.

[20] Reed L.J., Muench H. (1938). A simpe method of estimating fifity percent endpoints. Am. J. Epidemiol..

[21] Lee D.-H., Kwon J.-H., Noh J.-Y., Park J.-K., Yuk S.-S., Erdene-Ochir T.-O., Lee J.-B., Park S.-Y., Choi I.-S., Lee S.-W. (2016). Pathogenicity of the Korean H5N8 highly pathogenic avian influenza virus in commercial domestic poultry species. Avian Pathol..

[22] Adlhoch C., Fusaro A., Kuiken T., Niqueux É., Staubach C., Terregino C., Muñoz Guajardo I., Baldinelli F., European Food Safety Authority, European Centre for Disease Prevention and Control and European Union Reference Laboratory for Avian Influenza (2020). Avian influenza overview May–August 2020. EFSA J..

[23] Verhagen J.H., Fouchier R.A., Lewis N. (2021). Highly Pathogenic Avian Influenza Viruses at the Wild–Domestic Bird Interface in Europe: Future Directions for Research and Surveillance. Viruses.

[24] Sakuma S., Uchida Y., Kajita M., Tanikawa T., Mine J., Tsunekuni R., Saito T. (2021). First Outbreak of an H5N8 Highly Pathogenic Avian Influenza Virus on a Chicken Farm in Japan in 2020. Viruses.

[25] Khalil A., Fujimoto Y., Kojima I., Esaki M., Ri K., Masatani T., Matsui T., Ozawa M. (2021). Genetic Characterization of H5N8 Highly Pathogenic Avian Influenza Viruses Isolated from Falcated Ducks and Environmental Water in Japan in November 2020. Pathogens.

[26] Isoda N., Twabela A.T., Bazarragchaa E., Ogasawara K., Hayashi H., Wang Z.-J., Kobayashi D., Watanabe Y., Saito K., Kida H. (2020). Re-Invasion of H5N8 High Pathogenicity Avian Influenza Virus Clade 2.3.4.4b in Hokkaido, Japan, 2020. Viruses.

[27] Hill S.C., Lee Y.-J., Song B.-M., Kang H.-M., Lee E.-K., Hanna A., Gilbert M., Brown I.H., Pybus O.G. (2015). Wild waterfowl migration and domestic duck density shape the epidemiology of highly pathogenic H5N8 influenza in the Republic of Korea. Infect. Genet. Evol..

[28] Beerens N., Germeraad E.A., Venema S., Verheij E., Pritz-Verschuren S.B., Gonzales J.L. (2021). Comparative pathogenicity and environmental transmission of recent highly pathogenic avian influenza H5 viruses. Emerg. Microbes Infect..

