# Supplementary material for: Pathogenicity of H5N8 High Pathogenicity Avian Influenza Virus in Chickens and Ducks from South Korea in 2020–2021
Source: Viruses. 2021 Sep 23;13(10):1903. doi: 10.3390/v13101903 (PMC8539906; doi:10.3390/v13101903)
Supplement: Supplementary file 1 [file viruses-13-01903-s001.zip › viruses-1374140-supplementary.pdf]

Table S1 Virus titers in organs of individual chickens inoculated with H242/20(H5N8) and HD1/17(H5N6)

Virus	chicken	Virus titer[a] (log10 $TCID_{50}$/0.1ml, mean±SD)											
		Tra	Thy	Liv	Mus	Pro	Pan	SPL	CT	Lung	Kid	Heart	Brain
H242/20 (H5N8)	1	4.5	4.0	4.7	4.5	4.0	4.5	4.0	4.7	5.2	4.7	4.7	3.3
	2	4.8	3.7	3.7	3.7	4.7	2.5	2.7	4.5	4.7	4.5	3.7	4.3
	3	5.7	6.0	4.5	3.7	5.0	5.3	3.5	4.7	6.3	5.5	4.7	4.5
	Total	5.0±0.6	4.6±1.3	4.3±0.5	3.9±0.5	4.6±0.5	4.1±1.5	3.4±0.7	4.6±0.1	5.4±0.9	4.9±0.5	4.3±0.6	4.1±0.6
HD1/17 (H5N6)	1	5.7	8.5	8.5	5.7	8.5	4.4	8.5	8.5	8.5	7.8	8.5	4.8
	2	4.2	7.8	6.2	4.8	7.5	4.7	8.4	8.5	8.4	7.2	8.5	4.8
	3	6.3	8.0	6.7	5.5	5.5	6.7	6.3	6.5	8.5	8.4	8.4	8.4
	Total	5.4±1.1	8.1±0.3	7.1±1.2	5.3±0.5	7.2±1.5	5.2±1.3	7.7±1.2	7.8±1.2	8.5±0.1	7.8±0.6	8.5±0.1	6.0±2.1

Tra, trachea; Thy, thymus; Liv, liver; Mus, muscle; Pro, proventriculus; Pan, pancreas; SPL, spleen; CT, cecal tonsil; Kid,kidney. SPF chickens were inoculated with H242/20(H5N8) and HD1/17(H5N6) virus via the intranasal route at the titer of $10^6 EID_{50}$/0.1ml. Tissues were collected at 3 days post inoculation (n=3). [a]The virus titers were measure from the 10% homogenated tissues.

Table S2 Virus titers in organs of individual ducks inoculated with H242/20(H5N8) and HD1/17(H5N6)

Virus	bird	Virus titer[a] (log10 $TCID_{50}$/0.1ml, mean±SD)											
		Tra	Thy	Liv	Mus	Pro	Pan	SPL	CT	Lung	Kid	Heart	Brain
H242/20 (H5N8)	1	-	-	-	-	-	-	-	-	-	-	-	-
	2	-	-	-	-	-	-	-	-	-	-	-	-
	3	2.3	2.3	1.0	1.5	0.7	-	1.7	1.0	5.2	2.0	0.7	-
		2.3	2.3	1.0	1.5	0.7	-	1.7	1.0	5.2	2.0	0.7	-
HD1/17 (H5N6)	1	1.5	-	-	-	2.4	2.5	-	-	-	-	-	-
	2	-	-	-	-	2.2	1.5	-	-	-	-	-	-
	3	1.8	2.3	-	-	2.3	1.5	-	-	-	-	-	-
		1.7±0.2	2.3	-	-	2.3±0.1	1.8±0.6	-	-	-	-	-	-

Tra, trachea; Thy, thymus; Liv, liver; Mus, muscle; Pro, proventriculus; Pan, pancreas; SPL, spleen; CT, cecal tonsil; Kid,kidney. Ducks were inoculated with H242/20(H5N8) and HD1/17(H5N6) virus via the intranasal route at the titer of $10^6 EID_{50}$/0.1ml. Tissues were collected at 3 days post inoculation (n=3). [a]The virus titers were measure from the 10% homogenated tissues.

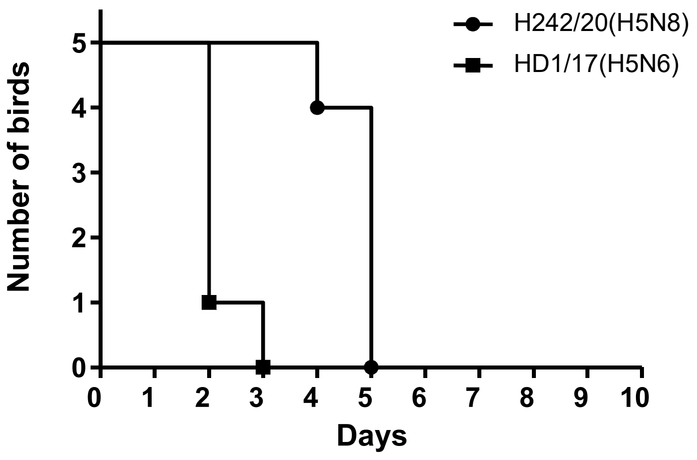

Figure S1. Survival curve of chickens inoculated with H242/20(H5N8) and HD1/17(H5N6). SPF chickens were inoculated with H242/20(H5N8) and HD1/17(H5N6) virus via the intranasal route at the titer of 10^6EID_{50}/0.1ml.
